# Hatching Time and Alevin Growth Prior to the Onset of Exogenous Feeding in Farmed, Wild and Hybrid Norwegian Atlantic Salmon

**DOI:** 10.1371/journal.pone.0113697

**Published:** 2014-12-01

**Authors:** Monica Favnebøe Solberg, Per Gunnar Fjelldal, Frank Nilsen, Kevin Alan Glover

**Affiliations:** 1 Population genetics research group, Institute of Marine Research, Nordnes, Bergen, Norway; 2 Reproduction and development biology research group, Matre Research Station, Institute of Marine Research, Matredal, Norway; 3 Sea lice Research Centre, Department of Biology, University of Bergen, Bergen, Norway; The Ohio State University, United States of America

## Abstract

The onset of exogenous feeding, when juveniles emerge from the gravel, is a critical event for salmonids where early emergence and large size provide a competitive advantage in the wild. Studying 131 farmed, hybrid and wild Norwegian Atlantic salmon families, originating from four wild populations and two commercial strains, we investigated whether approximately 10 generations of selection for faster growth has also resulted in increased somatic growth prior to the onset of exogenous feeding. In addition, we tested whether relaxed selection in farms has allowed for alterations in hatching time between farmed and wild salmon. Across three cohorts, wild salmon families hatched earlier than farmed salmon families, while hybrid families displayed intermediate hatching times. While the observed differences were small, i.e., 1–15 degree-days (0–3 days, as water temperatures were *c.* 5–6°C), these data suggest additive genetic variation for hatching time. Alevin length prior to exogenous feeding was positively related to egg size. After removal of egg size effects, no systematic differences in alevin length were observed between the wild and farmed salmon families. While these results indicate additive genetic variation for egg development timing, and wild salmon families consistently hatched earlier than farmed salmon families, these differences were so small they are unlikely to significantly influence early life history competition of farmed and wild salmon in the natural environment. This is especially the case given that the timing of spawning among females can vary by several weeks in some rivers. The general lack of difference in size between farmed and wild alevins, strongly suggest that the documented differences in somatic growth rate between wild and farmed Norwegian Atlantic salmon under hatchery conditions are first detectable after the onset of exogenous feeding.

## Introduction

Interactions between domesticated species and their wild conspecifics is a topic of concern in a variety of taxa including salmonids. Successful introgression of farmed Atlantic salmon *Salmo Salar* L. in wild populations has been documented in several rivers in several regions [1]–[3]. However, the degree of introgression due to farmed escapees or stocking with non-local hatchery strains seems to vary both in time and space. Recent spatio-temporal analyses of more than twenty Atlantic salmon populations throughout Norway revealed that detection of escaped salmon in native populations is not synonymous with introgression [4], [5]. While this could be caused by multiple reasons, density of the wild population has been suggested as a strong regulating factor [4], [6], [7]. This is because farmed and hatchery-reared salmonids have been documented to be competitively inferior to wild salmonids in terms of spawning success [8]–[10], and offspring survival in the wild [8], [11]–[13]. Thus, in cases where farmed or hatchery reared-salmonids have successfully spawned with wild conspecifics, the degree of admixture remaining in the wild population will be influenced by additive genetic variation in traits affecting the competitive balance between wild salmonids and their farmed/hatchery-reared conspecifics. Therefore, elucidating genetic differences between wild and farmed salmon is important in order to understand the ecological and evolutionary consequences of farmed salmon introgression in wild populations.

Commercial production of Atlantic salmon was initiated in Norway in the late 1960's [14] and breeding programs have included directional selection for a range of commercially important traits, including somatic growth. When farmed and wild salmon have been reared under identical hatchery conditions, significantly higher growth rates have been documented in farmed salmon [15]–[18]. These increased growth rates have been linked with increased appetite and feed consumption [19], in addition to more efficient utilisation of feed [20]. Genetic variation for both traits have been documented [21] and feed utilisation has furthermore been documented to be positively correlated to growth rate [20], [21]. Therefore, although not explicitly included in the selection programs, directional selection for increased growth over approximately ten generations is likely to have selected for both increased consumption and conversion efficiency.

Genes associated with the growth-regulation pathway, e.g., insulin growth factor-1 (IGF-1), have been documented to be similar [22] as well as to be up-regulated in farmed relative to wild salmonids after the onset of exogenous feeding [23]–[25]. Furthermore, alterations in transcription levels of genes associated with energy metabolism, which may influence growth, have been detected in farmed relative to wild Atlantic salmon alevins, i.e., small fish prior to the onset of exogenous feeding [26]. Whether directional selection for fast growth in farmed Norwegian salmon has resulted in increased growth rate both prior to and post the onset of exogenous feeding remains to be elucidated however. If salmon of farmed origin utilise endogenous resources more efficiently than salmon of wild origin, alevins emerging from eggs of farmed origin could potentially hold a temporary competitive size-advantage (e.g., in the context of successfully gaining and holding a territory) compared to alevins of wild origin, when everything else is equal (e.g., egg size, time of emergence and location).

Within salmonids, mortality in the wild is high at the juvenile stages. A critical event is the onset of exogenous feeding, when alevins emerge from the gravel [27]–[29], and both large and early emerging offspring have a competitive advantage [30]. At emergence, competition for residency and nutritional resources is high and phenology, i.e., timing of key life history stages with optimal seasonal environmental conditions, is crucial. Thus, spawning time in wild populations is adapted to the rivers thermal regime in order to secure optimal time of hatch and alevin emergence [31]. In contrast, relaxed selection, i.e., the reduction in natural selection pressure in the domestic environment may have allowed for both early and late onset of spawning, or prolonged spawning time, i.e., increased trait variance. Both similar [32] and deviating [33] embryonic developmental rates, have been documented in farmed and wild salmon of the Northwest Atlantic. When different, farmed salmon consistently hatched later than the wild salmon, while hybrids displayed intermediate hatching times [33]. This indicates that even if spawning time is similar, time of hatch and emergence of farmed alevins could be maladaptive to the natural environment, resulting in a competitive disadvantage which again may work as a potential barrier against introgression. How the process of domestication may have affected embryonic development rate, and thus hatching time, in Atlantic salmon originating from other strains and regions, such as the Northeast Atlantic where Atlantic salmon farming first was initiated, remains to be studied.

The present study aimed to investigate hatching time, as a proxy for embryonic development rate, and alevin growth in farmed, hybrid and wild salmon from Norway, by comparing cumulative degree-days from fertilisation to hatch and alevin length prior to the onset of exogenous feeding. Utilisation of endogenous resources was explicitly tested by the inclusion of maternal half-sibling strains of farmed and wild paternal origin. This was conducted on a total of 131 Atlantic salmon families, over three cohorts. Our main objectives were to investigate if approximately ten generations of domestication selection has resulted in i) alterations in embryonic development rate and thus time of hatch; and ii) increased somatic growth rate prior to the onset of exogenous feeding.

## Materials and Methods

### Overall design

In order to investigate hatching time (as a proxy for embryonic development rate) and alevin growth, in salmon of farmed, hybrid and wild origin, three cohorts of Norwegian Atlantic salmon families were studied from fertilisation to close to yolk sac absorption. Cumulative degree-days from fertilisation to hatch were documented and individual size measurements were collected prior to the onset of exogenous feeding. Alevin length at termination was then investigated with respect to egg sizes at the eyed-egg stage and cumulative degree-days post hatch.

### Strains

**Figure 1:**
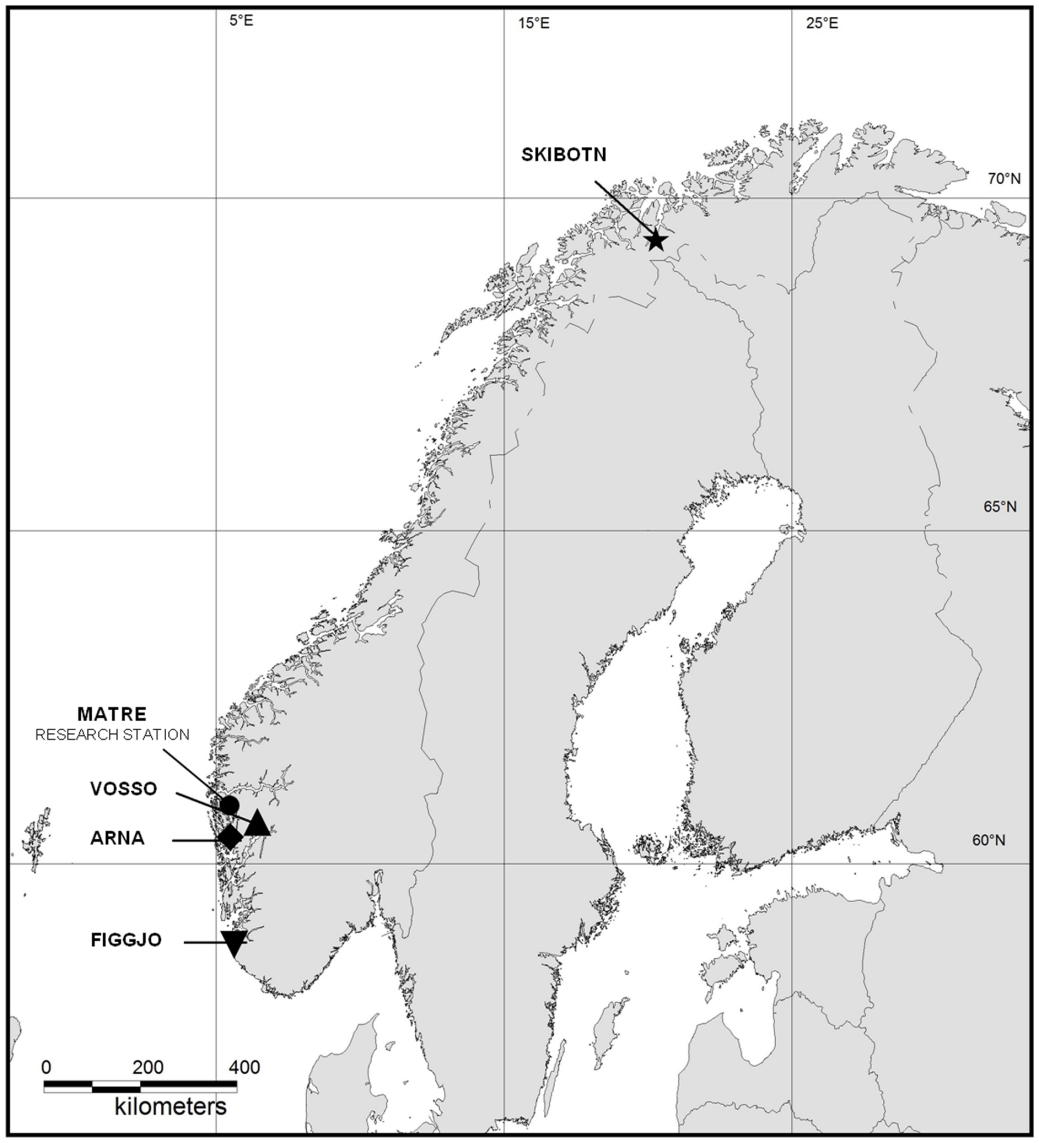
Location of wild salmon populations and the Matre research station. Wild salmon populations of four Norwegian rivers were included in the present study conducted at the Matre research station. Parental salmon were collected directly from the rivers, except for salmon of the River Skibotn strain that is conserved and reared in freshwater at the Norwegian Gene Bank for Atlantic salmon.

Parental salmon from four Norwegian wild populations (Figure 1) and two commercially farmed strains were used to generate nine experimental strains for this study. This comprised of four pure wild strains, two pure farmed strains and three farmed/wild F_1_ hybrid strains.

Wild salmon from the River Figgjo (58°81′N, 5°55′E), with a catchment area of *c*. 543 km^2^ [34], represents one of the largest salmon populations in Western Norway. This population consists mainly of one-sea-winter fish [35]. Parental salmon were caught by angling in the river, and temporarily transferred to a local hatchery. Later, these salmon were transported to and thereafter stripped for gametes at the Matre research station, where all experimental strains used in this study were established (Figure 1).

The River Arna (60°42′N, 5°46′E) is a small river located in Western Norway, and has a catchment area of *c*. 61 km^2^ [34]. The spawning population consists of salmon of varying winters at sea. Wild parental salmon were caught by a salmon trap located in the upper reaches of the watercourse, and were transferred to the local hatchery. Salmon were stripped in the hatchery, and unfertilised gametes were transferred to the Matre research station on the same day in order to create family groups for this study.

The River Vosso (60°64′N, 5°95′E) with a catchment area of *c*. 1 530 km^2^ [34] is the second largest watershed in Western Norway. This river is known for its large multi-sea-winter fish [36]. Due to a severe decline in the population since the early 1990's and an increase in farmed escapees at the spawning ground [36], this population, through maintenance of wild salmon and their offspring without any form of directional selection, has been conserved by the Norwegian Gene Bank for wild Atlantic salmon. The adult Vosso salmon used to create family crosses in the present study, originate from the gene bank, but had been reared from eggs to the smolt stage in the Voss hatchery before being released into the sea. These fish thereafter migrated with wild salmon into the ocean feeding grounds before returning to the River Vosso to spawn. Upon entry to the River Vosso, these fish were caught by angling or nets, and held in the Voss hatchery until stripping. Unfertilised gametes were collected from adults that were stripped in the Voss hatchery, and transported to the Matre research station on the same day to establish family groups.

The River Skibotn (69°38′N, 20°26′E), with a catchment area of *c*. 1181 km^2^ [34] is located in Northern Norway. Due to repeated infestation of the parasitic monogenean *Gyrodactylus salari*, this salmon population has been conserved by the Norwegian Gene Bank for Atlantic salmon (in a similar manner to the River Vosso population). Gametes were collected from spawners reared in the gene bank. Unfertilised gametes were transported to the Matre research station. Due to transportation time, gametes were fertilised approximately 24 hours post stripping.

The commercial Mowi strain from Marin Harvest was established in 1969, and is the oldest Norwegian farmed strain [14]. This strain was established from large multi-sea winter fish collected from the River Bolstad in the Vosso watercourse and the River Åroy, in addition to wild salmon caught in the sea outside of Western Norway, near Osterfjord and Sotra [37], [38]. Through an approximate four-year generation cycle, this strain has been selected for increased growth, delayed maturation and fillet quality [38]. Offspring of the 9^th^ and 10^th^ generation were used as parents for this experiment. Unfertilised gametes were collected from the breeding station at Askøy, Western Norway, and transported to the Matre research station on the same day.

The SalmoBreed strain was commercially established in 1999, and is based upon genetic material from several Norwegian farmed strains that has been under commercial selection since the late 1960's and early 1970's. Offspring of the approximately 10^th^ generation of selected parents were used to generate the biological material in this study. Gametes were collected at one of the SalmoBreed breeding stations, located on Osterøy, Western Norway, and transported to the Matre research station on the same day.

### Production of experimental strains

Three experimental cohorts, consisting of 29, 39 and 62 families of wild, farmed and F_1_ hybrid origin were established on November 23, 2010, November 16 and 22–23, 2011, and November 12–15, 2012, respectively. Thus, a total of 131 families were produced across all three cohorts. The respective cohorts, hatched in spring the following year, are hereon called C2011, C2012 and C2013. Cohorts consisted of three, six and nine experimental strains respectively, where F_1_ hybrid strains were named by their pure maternal × paternal half-sibling strains. F_1_ hybrids were not created between all wild and farmed strains due to logistical reasons, i.e., limiting number of eggs available. The adjoining three or four maternal and paternal half-sibling strains, i.e., the pure strains of wild and farmed origin and their F_1_ hybrid strain(s), are in this study combined referred to as a wild/farmed cross. All gametes were fertilised upon arrival at the Matre Research station (Figure 1). Stripping dates were synchronised as far as practically possible within each cohort.

For C2011, ova and milt from wild salmon of the River Figgjo strain and from farmed salmon of the commercial Mowi strain were used to produce nine wild families, ten farmed families and ten F_1_ hybrid families, generated by crossing farmed females with wild males.

For C2012, gametes from wild salmon of the River Figgjo strain and from farmed salmon of the commercial Mowi strain were used to generate four experimental strains; six wild families, seven farmed families, seven F_1_ hybrid families generated by crossing farmed females with wild males, and six F_1_ hybrid families generated by crossing wild females with farmed males. In addition, gametes from salmon of the River Vosso strain were used to generate seven wild families, while ova and milt from wild salmon of the River Arna strain were used to generate a further six wild families. It was not possible to fully synchronize stripping of the Arna strain with the rest of the crosses; hence these families were produced one week earlier than the remaining families of C2012.

For C2013, gametes from wild salmon of the River Figgjo strain and from farmed salmon of the commercial Mowi strain were again used to generate four experimental strains. Using the same family design as in C2012; 9, 8, 9 and 7 families were generated per strain, respectively. Gametes from wild salmon of the River Vosso strain and from farmed salmon of the commercial SalmoBreed strain were used to generate three experimental strains; four wild families, eight farmed families and six F_1_ hybrid families generated by crossing farmed females with wild males. In addition, ova and milt from wild salmon of the River Arna strain were used to generate eight wild families, while gametes from salmon of the wild Skibotn strain, conserved by the Norwegian Gene bank for Atlantic salmon, were used to generate a further four wild families.

Biological information of all parental salmon used in this study is given in Table 1.

**Table 1:** Weight and length measurements of parental salmon of cohort 2011, 2012 and 2013.

					Weight (kg)		Fork length(cm)	
Cohort	Strain	Origin	Sex	*n*	Mean	Range	Mean	Range
2011	Figgjo	Wild	Female	9	2.14	1.64–2.94	66.18	60–72
			Male	8	1.98	1.05–2.75	61.7	51–71
	Mowi	Farm	Female	10	12–14	NA	NA	NA
			Male	10	14–18	NA	NA	NA
2012	Figgjo	Wild	Female	7	4.07	3.02–4.98	74	68–80
			Male	8	2.49	1.22–3.58	64.25	32–75
	Mowi	Farm	Female	8	12–14	NA	NA	NA
			Male	7	14–18	NA	NA	NA
	Vosso	Wild	Female	7	5.35	4.05–6.80	81.43	75–89
			Male	7	5.72	1.66–12.70	83.86	60–109
	Arna	Wild	Female	4	4.1	3.60–4.50	80.25	75–84
			Male	6	5.13	2.00–8.30	81.83	62–96
2013	Figgjo	Wild	Female	9	2.4	1.40–3.05	68.1	59 –76
			Male	9	2.81	1.79–5.38	68.4	59 –86
	Mowi	Farm	Female	9	12–14	NA	NA	NA
			Male	8	14–16	NA	NA	NA
	Vosso	Wild	Female	4	NA	NA	NA	NA
			Male	6	NA	NA	NA	NA
	SalmoBreed	Farm	Female	8	08–12	NA	NA	NA
			Male	7	12–14	NA	NA	NA
	Arna	Wild	Female	8	5.49	4.00–9.10	81.25	74–96
			Male	8	3.34	2.10–6.60	70.63	62–89
	Skibotn	Wild*	Female	2	11.39	9.64–13.13	90	85–95
			Male	2	6.28	5.89–6.67	81.5	78–85

Gametes from a total of 85 females and 86 males were used to generate the 131 Atlantic salmon families of farmed, hybrid or wild origin included in this study. Weight measurements of the farmed salmon are approximated. * Wild salmon population reared in freshwater at the Norwegian Gene Bank for Atlantic salmon.

### Experimental conditions

Fertilised eggs were incubated in single-family units fed from a single water source and water temperatures were allowed to fluctuate naturally throughout the incubation period. This gave mean water temperatures of 4.8°C (range 3.0°C–6.9°C), 6.0°C (4.2°C–8.4°C), and 6.3°C (5.5°C–7.8°C) for cohorts C2011, C2012 and C2013 respectively.

At the eyed-egg stage, all families were first shocked in order to sort out dead eggs. Mean egg diameter per family was measured by dividing the numbers of eggs aligned along a 25 cm linear egg holder. Thereafter, eggs were sorted into miniature hatchery trays containing up to 30 eggs per family. The 29 families of C2011 were represented in four replicates containing 25 eggs/replicate (116 units in total). The 39 families of C2012 were represented in three replicates containing 25 eggs/replicate (117 units in total), although two families unites were by a mistake provided with 26 eggs. The 63 families of the C2013 were represented in two replicates containing 30 eggs/replicate (126 units in total).

Once in miniature hatchery trays, eggs were photographed daily through the hatching period. Hatching success and cumulative degree-day to hatch were documented from the photographs, and half emerged alevins were classified as hatched, if fully emerged the next day.

The experiment was terminated prior to the onset of exogenous feeding, at the detection of close to fully absorbed yolk sacs. C2011 and C2013 were terminated across two days, while C2012 was terminated on a single day. All individuals were euthanised with an overdose of metacain (Finquel Vet, ScanVacc, Årnes, Norway). Ten individuals from each family replicate were photographed and total length was measured by the use of ImageJ v. 1.56 for Windows. Individual weight measurements were taken of all photographed individuals for C2011 and C2013. For C2012, only a bulk weight of each family replicate was taken. Thus, growth comparisons of alevins were performed based upon total length prior to the onset of exogenous feeding, as this measurement was considered the most accurate in all three cohorts.

### Ethics Statement

The experiments were performed in accordance with the general guidelines for animal studies, the Animal Research Reporting In Vivo Experiments (ARRIVE) guidelines [39].

Parental broodstock caught in the wild, i.e., in the River Arna, Vosso and Figgjo, were captured by local hatcheries doing supporting breeding in accordance with Norwegian regulations. No permits/licenses regarding the handling of the broodstock of this study were required by the research team. Adult Figgjo salmon were transported to the Matre Research station by a commercial company specialised in road transport of live fish, and later stripped at the research station by the research team. Stripping of wild salmon caught in the River Arna and River Vosso was performed by hatchery staff at the respective locations, with members of the research team assisting. Post stripping, gametes were transported directly to the Matre research station by members of the research team. In all three rivers catching and stripping of wild salmon were done for routine hatchery proposes. Transportation of adult individuals, i.e., Figgjo, and gametes, i.e., Arna and Vosso, to the Matre research station were done for the sole purpose of research.

Gametes collected at commercial breeding stations and in the Norwegian Gene bank for Atlantic salmon were collected following standard procedures at the respective locations. Salmon of the river Skibotn strain were stripped by the staff at the Norwegian Gene Bank for Atlantic salmon and gametes were shipped overnight to the Matre research station. Stripping of farmed salmon, i.e., Mowi and SalmoBreed, was performed by staff at the breeding stations. Members of the research team were present during stripping of farmed salmon, i.e., assisting in the collection of the gametes, before transporting them to the research station.

Parental salmon were anesthetised prior to stripping, and individuals that were terminated post stripping were euthanised with an overdose of metacain (Finquel Vet, ScanVacc, Årnes, Norway), followed by a sharp blow to the head. Scale samples were taken from all parental salmon caught in the wild, and analysed to ensure that they were not escapees from farms [40]. Scale samples were collected from euthanised individuals, in a rectangular area between the dorsal and the adipose fin, above the lateral line.

Fertilised eggs and alevins prior to exogenous feeding are exempted from the Norwegian Regulation on Animal Experimentation, and thus approval of the experimental protocol of this experiment by the Norwegian Animal Research Authority (NARA) was not needed. However, welfare and use of experimental animals were none the less performed in strict accordance with the Norwegian Animal Welfare Act. In addition, all personnel involved in the experiment had undergone training approved by the Norwegian Food Safety Authority, which is mandatory for all personnel running experiments involving animals included in the Animal Welfare Act.

### Statistical analyses

All statistical analyses were performed in R version 3.1.0. [41], with critical P-values set to 0.05.

In order to investigate the relationship between maternal size and egg size, as a proxy for per egg investment, a linear model was fitted. We first tested for effects of maternal body log-weight (*W*), maternal origin (*O*), and cohort (*C*), including all two-way interactions, upon mean egg diameter (*E*) at the eyed-egg stage: 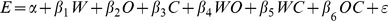
(1)where *ε* is a random error. The predictor variable maternal body weight was log transformed, as the relationship between egg diameter and maternal weight was not linear. i.e., after a certain body size egg size no longer increased. Model selection was performed by backward selection on the full model, based upon Akaike Information Criterion (AIC) values. Models displaying AIC values of ±2 were considered equally good, and by the principle of parsimony the simplest model was selected. For AIC comparisons of the linear models see File S1.

In order to investigate hatching time of farmed, hybrid and wild salmon, as a proxy for rate of embryonic development, linear mixed effects (LME) models were fitted using the *lmer* function in the lme4 package [42]. We tested for effects of mean family log egg diameter (*E*) and strain (*S*), as well as their interaction, upon log cumulative degree-days from fertilisation to hatch (*D*). The response variable, cumulative degree-days from fertilisation to hatch, and the predictor variable mean family egg diameter was log-transformed, due to the non-linear relationship between them [43]. Maternal identity (*dam*), and family (*f*) nested within strain and replicate (*r*) nested within family were included as random intercept factors: 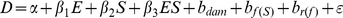
(2)where *ε* is a random error. Separate models were fitted for every cohort, due to variation in water temperature between the three experimental periods. Thus, three models were fitted in total.

LME models were also used to investigate the effect of mean family log egg diameter (*E*), strain (*S*) and median family replicate log degree-days post hatch (*D*), including all two-way interactions, upon total alevin length prior to the onset of exogenous feeding (*L*). Maternal identity (*dam*), family (*f*) nested within strain and replicate (*r*) nested within family were included as random intercept factors: 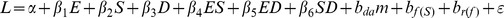
(3)where *ε* is a random error. Again, separate models were fitted for every cohort, i.e., three models in total.

Model selection of the LME models 2 and 3 was performed backwards by the use of the *step* function in the lmerTest package [44]. By this procedure, insignificant random effects were eliminated, followed by the removal of insignificant fixed effects. Interaction terms were removed before the variables themselves (if significant two-way interaction terms were detected both variables were included in the final model, regardless of their significance level). P-values for the random effects were calculated based upon likelihood ratio tests, while F-statistics, denominator degrees of freedom and P-values calculated based on Satterthwaite's approximations were presented for the fixed effects [44]. For the significant categorical fixed effects, least squares means and differences of least squares means were calculated, i.e., pair-wise parameter level tests.

Trait variance, i.e., within-strain variation in time of hatch and alevin length were calculated using coefficient of variance (CV  = 100× SD/mean).

## Results

### Egg size in relation to maternal body size

Eggs from a total of 85 females were used to generate the 131 families included in this study (Table 1). Growth measurements were not collected from the four females of the River Vosso strain used in the Vosso/SalmoBreed cross of C2013. In addition, the two females used to generate the River Skibotn strain of C2013 were excluded from the analysis due to the low sample size. Hence, a total of 79 females were included in the below analysis.

**Figure 2:**
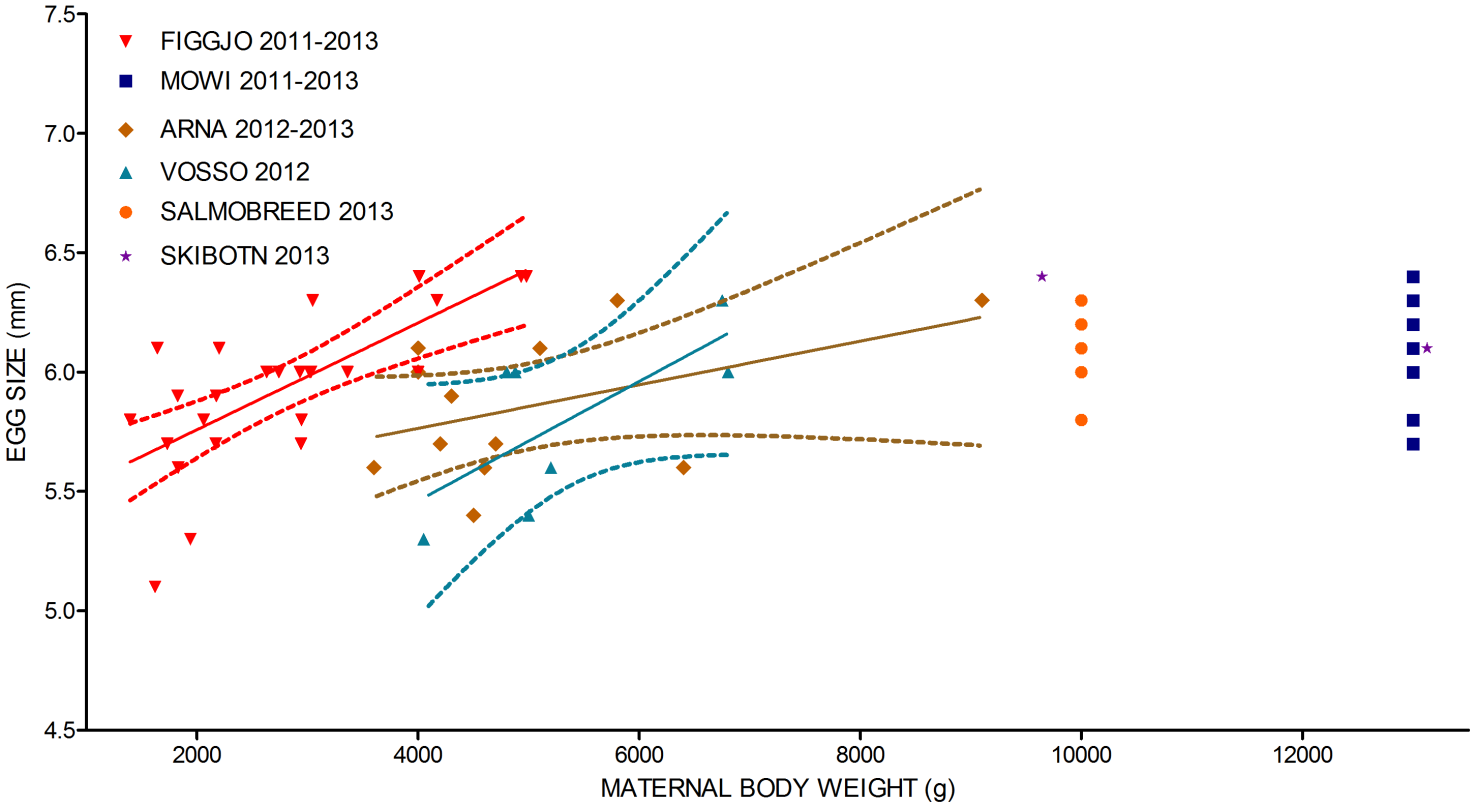
Egg size in relation to maternal body size. The effect of maternal body size upon mean egg sizes in salmon of farmed and wild origin, illustrated by the regression plot between mean egg sizes (mm in diameter) and maternal body weight (grams). Dotted lines illustrate the 95% confidence interval. For model selection of the linear model investigating the relationship between maternal log-weight and egg diameter, see File S1.

In general, larger females produced larger eggs (in diameter) than smaller females (F = 17.23, Df = 1, Sum Sq = 0.88, P<0.0001) (Figure 2). Thus, mean log-egg size increased with maternal log-body weight. As maternal body size was higher in the farmed strains, larger sized eggs were in general detected in the farmed salmon as compared to the wild salmon. However, the relationship between egg diameter and maternal body size was not linear and egg diameter did not increase in fish larger than approximately 6 kg (Figure 2).

A significant effect of origin upon egg size were also detected (F = 7.08, Df = 4, Sum Sq = 1.45, P<0.0001), hence maternal body size alone did not explain the between-strain variation in mean egg size. In the wild strains, the River Figgjo females produced larger eggs relative to their maternal body size than the River Arna and the River Vosso females (Figure 2). The two farmed strains, i.e., Mowi and SalmoBreed, produced smaller eggs relative to their maternal average size than the wild strains (Figure 2). Thus, per-egg investment, i.e., mean egg size relative to maternal body size, differed between the strains. This may, however, be contextual and influenced by the ambient environment experienced by the different maternal strains. No significant effect of cohort was detected in the analysis (File S1). Information about number of eggs produced per female were not available for most strains, hence we were not able to investigate overall fecundity in terms of both egg size and number of eggs.

### Time of hatch

**Figure 3:**
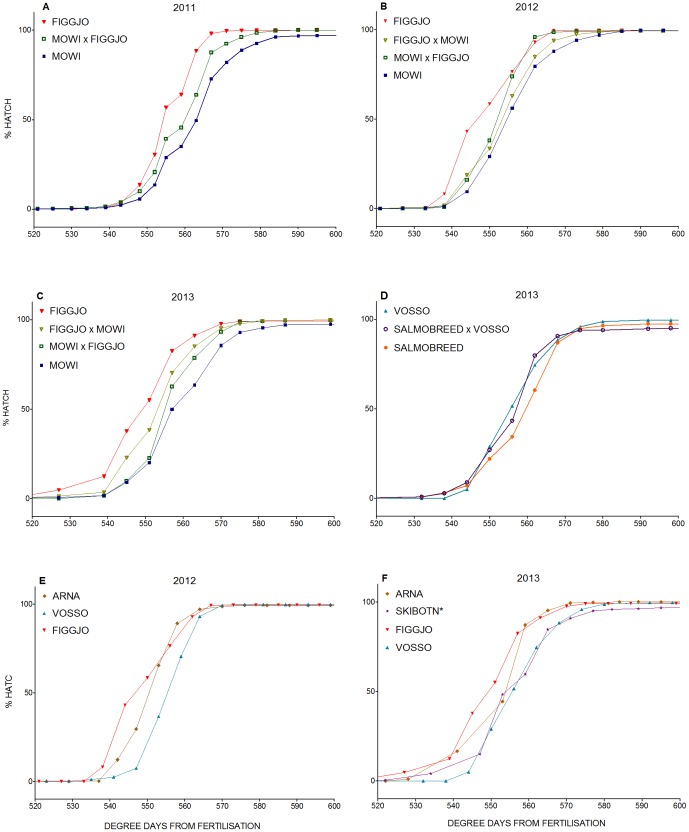
Embryonic development rate. Mean cumulative degree-days from fertilisation to percentage hatch in the; A) Figgjo/Mowi cross C2011, B) Figgjo/Mowi cross C2012, C) Figgjo/Mowi cross C2013, D) Vosso/SalmoBreed cross C2013, E) wild strains C2012, and F) wild strains C2013. See Table 3, 4 and File S2 for the adjoining statistical results.

Hatching success was very high in all three cohorts, i.e., 99, 99.4 and 98.7% for C2011, C2012 and C2013 respectively (Table 2, Figure 3). Hatching was observed between 521–601, 515–619, 489–677 degree-days post fertilisation, and mean cumulative degree-days to hatch of C2011, C2012 and C2013 were 563, 556 and 557 degree-days respectively (Table 2, Figure 3). No clear indication of dissimilarities in trait variance, i.e., coefficient of variation (CV) for time of hatch, was detected in salmon of farmed and wild origin (Table 2).

**Table 2:** Number, egg size, cumulative degree-days to hatch and total length prior to the onset of exogenous feeding in salmon of farmed, hybrid and wild origin of C2011, C2012 and C2013.

					Egg diameter (mm)			Cumulative degree-days to hatch				Not hatched		Length prior to start-feeding (mm)				
Cohort	Strain	Origin	Families	*n*	Median	Mean	Range	Median	Mean	Range	CV	n	%	*n*	Median	Mean	Range	CV
2011	Figgjo	Wild	9	900	5.7	5.78	5.3–6.1	555	557	534–590	1.22	0	0	360	28.7	28.7	26.4–30.8	2.85
	Mowi × Figgjo	F1 hybrid	10	1000	6	6	5.8–6.3	563	561	521–590	1.63	0	0	400	28.9	29	26.6–30.8	2.42
	Mowi	Farm	10	1000	6	5.97	5.8–6.1	563	564	525–601	1.73	29	2.9	400	29	29	26.2–31.5	2.49
2012	Figgjo	Wild	6	451	6.25	6.26	6.0–6.4	550	551	538–573	1.55	3	0.7	180	28.2	28.3	25.7–30.3	2.83
	Figgjo × Mowi	F1 hybrid	6	450	6.2	6.2	6.0–6.4	556	556	527–619	1.68	3	0.7	180	28.3	28.2	26.6–29.9	2.35
	Mowi × Figgjo	F1 hybrid	7	525	6.1	6.06	5.8–6.4	556	554	538–585	1.21	4	0.8	210	28.1	28.1	25.6–30.0	3.31
	Mowi	Farm	7	525	6.1	6.14	5.8–6.3	556	558	515–590	1.77	3	0.6	210	28.2	28.1	25.4–29.9	2.71
	Vosso	Wild	7	526	6	5.8	5.3–6.3	559	558	523–576	1.18	1	0.2	210	27.4	27.4	25.3–29.5	2.97
	Arna	Wild	6	450	5.7	5.72	5.4–6.1	553	553	537–576	1.22	3	0.7	180	28.4	28.4	26.9–30.3	2.92
2013	Figgjo	Wild	9	540	6	5.86	5.0–6.3	551	552	514–587	1.87	4	0.7	180	27.7	27.8	25.1–30.1	3.48
	Figgjo × Mowi	F1 hybrid	7	420	6	5.89	5.2–6.3	557	556	524–599	1.75	1	0.2	140	28	27.9	25.2–30.0	3.63
	Mowi × Figgjo	F1 hybrid	9	540	6.1	6.12	5.7–6.4	557	559	539–599	1.56	3	0.6	180	28.6	28.5	25.1–30.6	3.24
	Mowi	Farm	8	480	6.1	6.11	5.8–6.4	557	563	489–677	2.61	4	0.8	160	28.4	28.3	25.2–30.8	3.7
	Vosso	Wild	4	240	5.9	5.88	5.7–6.0	556	559	544–592	1.6	2	0.8	80	28.3	28.3	26.5–30.0	2.61
	SalmoBreed × Vosso	F1 hybrid	6	360	6	6.03	5.8–6.3	562	558	501–604	1.65	17	4.7	120	28.1	28	25.9–29.4	2.15
	SalmoBreed	Farm	8	480	6.1	6.1	5.8–6.4	562	561	519–675	2.03	10	2.1	160	28.2	28.2	26.7–29.9	2.38
	Arna	Wild	8	480	5.85	5.91	5.6–6.3	553	548	507–577	2.3	1	0.2	160	27.5	27.6	24.7–30.2	3.85
	Skibotn	Wild*	4	240	6.25	6.25	6.1–6.4	559	558	515–607	1.94	7	2.9	80	27.8	27.6	24.7–29.1	3.76

CV  =  coefficient of variation.*Wild salmon population reared in freshwater at the Norwegian Gene Bank for Atlantic salmon.

Significant differences in hatching time, i.e., cumulative log degree-days from fertilisation to hatch, were detected between strains of farmed, hybrid and wild origin, in all three cohorts (Table 3, 4 and File S2). In all cohorts, wild eggs consistently hatched earlier than farmed eggs (Table 2, Figure 3), with hybrids displaying intermediate hatching times within the wild/farmed crosses (Table 2, Figure 3a–d). Within the Figgjo/Mowi crosses, a significant difference in hatching time was detected between the Figgjo (wild) and Mowi strains (farmed) in all three cohorts (Table 4). The difference in hatching time between F_1_ hybrid strains and their maternal/paternal half-sibling strains, i.e., Figgjo or Mowi, were also significant in some of the cohorts (Table 4). Within the Vosso/SalmoBreed cross, included only in C2013, wild salmon eggs hatched earlier than farmed salmon eggs, however, this was not statistically significant (Table 4). The farmed SalmoBreed strain did however hatch later than some of the other wild strains in C2013 (Table 4). Significant effects of origin upon cumulative degree-days to hatch was also detected between some of the wild strains, e.g., the wild Arna hatched earlier than all other strains in C2013 except one (Table 4).

**Table 3:** Significance levels of random and fixed effects included in the full linear mixed effect models (model 2) investigating variation in cumulative log degree-days from fertilisation to hatch in salmon of farmed, hybrid and wild origin of C2011, C2012 and C2013.

					Random effects				Fixed effects					
Cohort	*n* Strains	*n* Families	*n*	Response	Variable	Chi.sq	Chi.Df	P	Variable	Sum.sq	NumDf	DenDf	F	P
2011	3	29	2871	Log-D	Dam	13.1	1	0.0003	Strain × log-egg	0.0	2	12.36	0.12	0.88
					Strain/Family	9.5	1	0.002	Log-egg	0.0	1	21.13	0.0002	0.99
					Family/Rep	104.3	1	<0.0001	Strain	0.0003	2	12.53	6.13	**0.01**
2012	6	39	2910	Log-D	Dam	4.3	1	0.04	Strain × log-egg	0.0	5	18.06	0.17	0.97
					Strain/Family	8.1	1	0.004	Log-egg	0.0	1	28.94	2.35	0.13
					Family/Rep	271.5	1	<0.0001	Strain	0.0003	5	17.39	2.88	**0.046**
2013	9	63	3731	Log-D	Dam	1.47	1	0.23	Strain × log-egg	0.0005	8	108.04	1.38	0.21
					Strain/Family	0.0	1	1	Log-egg	0.0010	1	116	5.05	**0.027**
					Family/Rep	976.52	1	<0.0001	Strain	0.0021	8	116	5.47	**<0.0001**

One model was fitted per cohort. *n:* number of individuals included in the analysis, i.e., after mortality. Random effects: Dam; maternal identity. Strain/Fam; family nested within strain (9–10, 6–7 and 4–9 families per strain in C2011, C2012 and C2013, respectively), Family/Rep; replicate nested within family (4, 3 and 2 replicates per family in C2011, C2012 and C2013, respectively). Chi.sq; value of the Chi square statistics. Chi.Df; the degrees of freedom for the test. P; p-value of the likelihood ratio test for the random effect. Fixed effects: Strain; Mowi (C2011–C2013), Mowi × Figgjo (C2011–2013), Figgjo (C2011–2013), Figgjo × Mowi (C2012–2013), Vosso (C2012–2013), Arna (C2012–2013), SalmoBreed (C2013), SalmoBreed × Vosso (C2013), Skibotn (C2013). Log-egg; mean family log egg diameter. Strain × log-egg; interaction between strain and egg size. Sum.sq; sums of squares. Num.Df; numerator degrees of freedom. Den.Df; denominator degrees of freedom (Satterthwaite's approximation) F; F-value. P; P-value. Significant fixed effects are marked in bold. For differences in least squares means between strains of C2011, C2012 and C2013, see File S2.

**Table 4:** P-values of the parameter level tests of the final LME models (model 2) investigating strain difference in time of hatch in salmon of farmed, hybrid and wild origin of C2011, C2012 and C2013.

	Mowi (M)			SalmoBreed (S)	M♀ × F♂			F♀ × M♂		S♀ × V♂	Figgjo (F)		Vosso (V)		Arna
	C2011	C2012	C2013	C2013	C2011	C2012	C2013	C2012	C2013	C2013	C2012	C2013	C2012	C2013	C2013
SalmoBreed			0.5												
M♀ × F♂	**0.02**	**0.03**	0.2	0.5											
F♀ × M♂		0.3	**0.04**	0.2		0.6	0.5								
S♀ × V♂			0.09	0.3			0.7		0.8						
Figgjo	**0.01**	**0.02**	**0.0002**	**0.002**	0.2	0.4	**0.01**	0.06	0.08	0.06					
Vosso		0.8	0.5	0.9		0.2	0.7	0.4	0.3	0.4	**0.03**	**0.01**			
Arna		0.08	**<0.0001**	**<0.0001**		0.7	**<0.0001**	0.4	**0.001**	**0.0008**	0.7	0.09	0.1	**0.0002**	
Skibotn			0.09	0.3			0.6		0.9	0.9		0.2		0.4	**0.007**

M♀ × F♂: Mowi × Figgjo, F♀ × M♂: Figgjo × Mowi, S♀ × V♂: SalmoBreed × Vosso. P-values indicate the differences of least squares means of cumulative log degree-days from fertilisation to hatch between strains. See File S2 for more statistical outputs. Significant results are marked in bold.

A positive relationship between mean family log-egg diameter and cumulative log degree-days to hatch was detected in C2013 (Table 3). This relationship was very weak however, and the slope of the relationship indicates that time of hatch increased with approximately 1 degree-day per 0.1 mm increase in egg diameter.

Although significant effects of origin upon cumulative degree-days to hatch were detected in this study, the actual differences in time of hatch between farmed, wild and F_1_ hybrid strains were small. The largest differences in mean cumulative degree-days until hatch between wild and farmed strains were 7, 7 and 15 degree-days for C2011, C2012 and C2013 respectively. For maternal half-siblings, the corresponding numbers were 3, 5 and 4 degree-days respectively. Considering the fact that mean time of hatch in all cohorts were>550 degree-days post fertilisation, this indicates that differences in embryonic development rate between wild and farmed salmon in this study were typically 1–2%.

### Alevin growth prior to the onset of exogenous feeding

**Figure 4:**
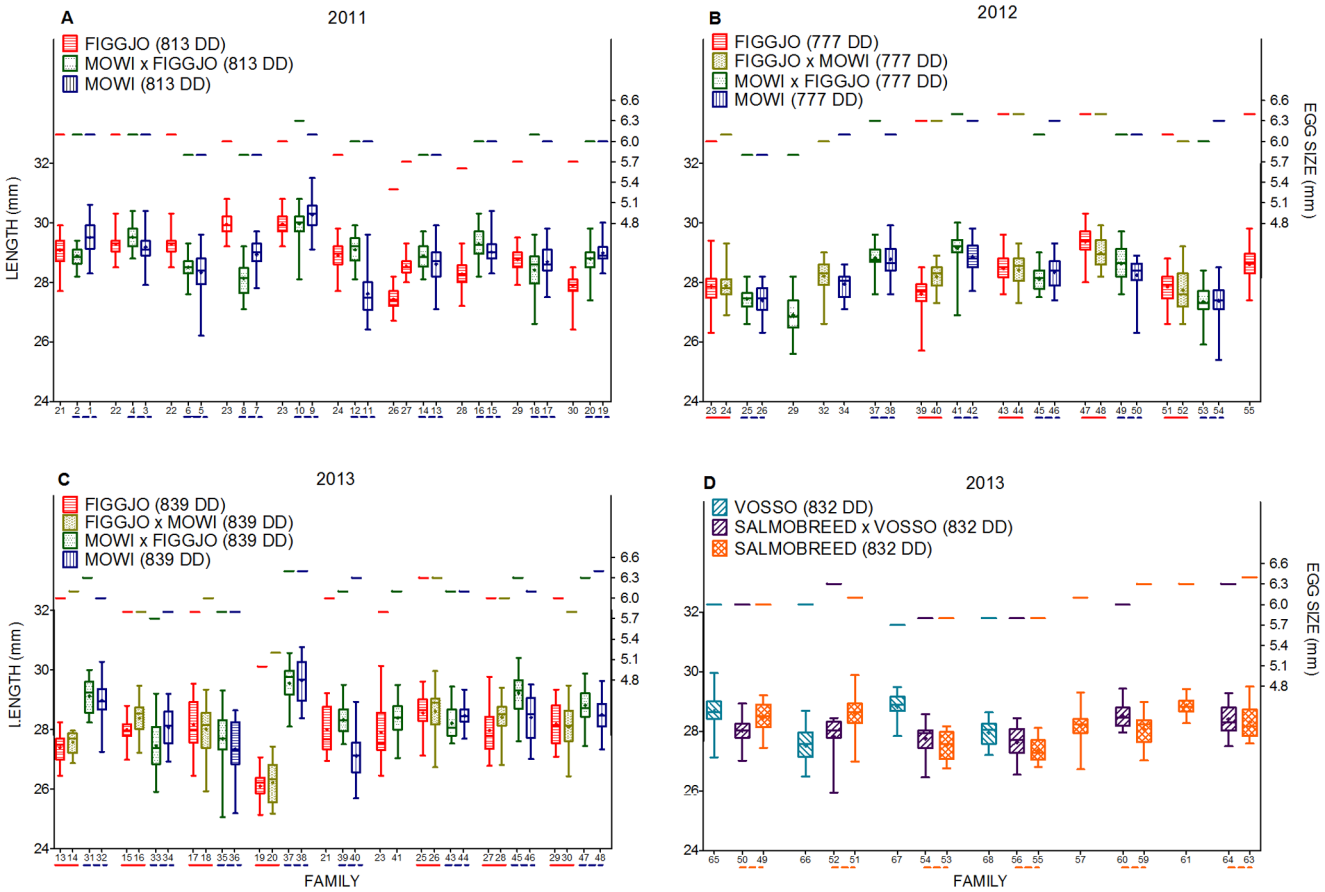
Egg size and alevin length prior to the onset of exogenous feeding in wild/farmed crosses. A) Figgjo/Mowi cross C2011, B) Figgjo/Mowi cross C2012, C) Figgjo/Mowi cross C2013 and D) Vosso/SalmoBreed cross C2013. DD: degree-days from fertilisation to termination prior to the onset of exogenous feeding. Families are sorted in groups containing their respective maternal and paternal half-sibling, i.e., three families per group in Figure A and D, and four families per groups in Figure B and C. Wild/farmed hybrids are named by the origin of their maternal × paternal half-siblings. Maternal half-siblings emerging from farmed eggs are illustrated by dotted lines, while maternal half-siblings emerging from wild eggs are illustrated by solid lines. In C2011, F_1_ hybrid family 4 and 6 are paternal half-siblings to Figgjo family 22, while F_1_ hybrid family 8 and 10 are paternal half-siblings to Figgjo family 23. In addition, Figgjo family 26 and 27 is paternal half-siblings to F_1_ hybrid family 14. Boxes show the median (thick line), mean (+), 1^st^ and 3^rd^ quartiles (lower and upper boundary) and the lower and upper extreme (whiskers). See Table 5 for the adjoining statistical results.

**Figure 5:**
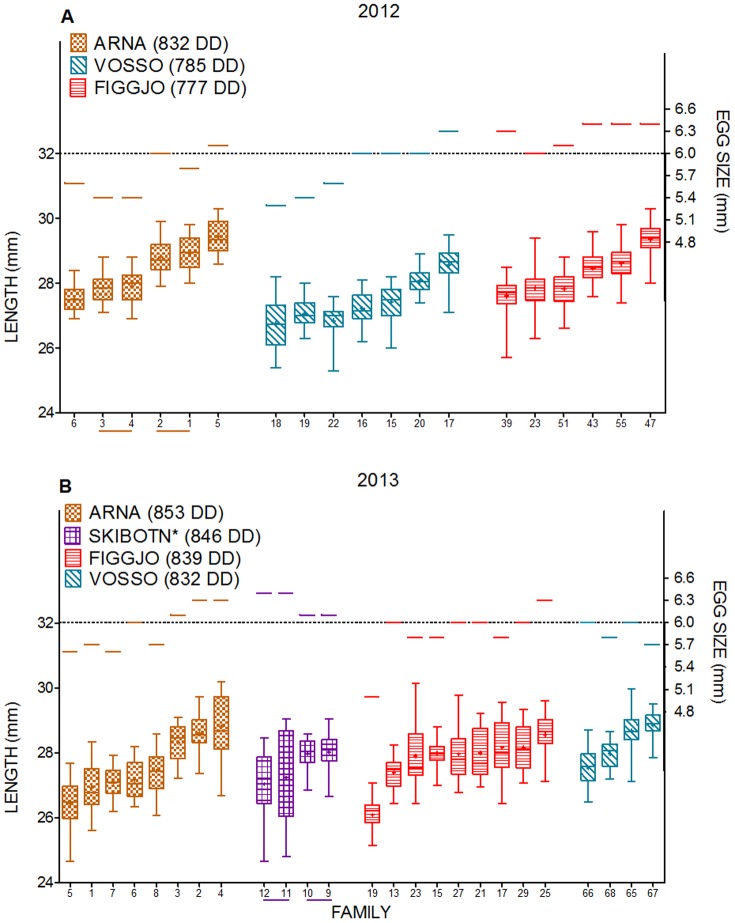
Egg size and alevin length prior to the onset of exogenous feeding in wild strains. A) C2012 and B) C2013. DD: degree days from fertilisation until termination. Strains are sorted by cumulative degree day from fertilisation to termination prior to the onset of exogenous feeding, while families within each strain are sorted by their length, both in increasing order. Maternal half-siblings are illustrated by solid lines. In C2013, Skibotn family 9 and 11, and 19 and 12 are paternal half-siblings. Boxes show the median (thick line), mean (+), 1^st^ and 3^rd^ quartiles (lower and upper boundary) and the lower and upper extreme (whiskers). See Table 5 and File S2 for the adjoining statistical results.

Alevin length was measured prior to the onset of exogenous feeding, i.e., 810–816, 777–832, 832–853 degree-days post fertilisation in C2011, C2012 and C2013 respectively (Table 2, Figure 4 and 5). Cohorts were terminated across a maximum of two days, thus differences in degree-days post fertilisation were primarily due to the differences in fertilisation dates.

An overall positive relationship between alevin length and mean family log egg size was detected in all cohorts (Table 5, Figure 4 and 5). The slope of this relationship indicates that alevin length increased with approximately 0.21 mm, 0.14 mm and 0.16 mm per 0.1 mm increase in egg diameter in C2011, C2012 and C2013, respectively. Thus, a stronger effect of egg size was detected in C2011, where the mean water temperature was >1.2°C lower than in C2012 and C2013.

**Table 5:** Significance levels of random and fixed effects included in the full linear mixed effect models (model 3) investigating variation in total length prior to the onset of exogenous feeding in salmon of farmed, hybrid and wild origin of C2011, C2012 and C2013.

					Random effects				Fixed effects					
Cohort	*n* Strains	*n* Families	*n*	Response	Variable	Chi.sq	Chi.Df	P	Variable	Sum.sq	NumDf	DenDf	F	P
2011	3	29	1160	L (mm)	Dam	1.9	1	0.2	Strain × log-egg	0.01	2	22.61	0.02	0.98
					Strain/Family	54.0	1	<0.0001	Strain × log-D	0.20	2	104.10	0.67	0.51
					Family/Rep	67.8	1	<0.0001	Log-D × log-egg	0.00	1	110.10	0.00	0.99
									Strain	0.80	2	25.57	0.77	0.47
									Log-D	2.40	1	111.65	16.42	**0.0001**
									Log-egg	8.55	1	26.99	46.59	**<0.0001**
2012	6	39	1170	L (mm)	Dam	26.5	1	<0.0001	Strain × log-egg	0.29	5	36.11	0.24	0.94
					Strain/Family	0.8	1	0.4	Strain × log-D	2.60	5	126.25	2.14	0.07
					Family/Rep	55.0	1	<0.0001	Log-D × log-egg	0.02	1	271.24	0.09	0.77
									Strain	4.25	5	27.41	2.35	0.07
									Log-D	1.02	1	304.40	6.88	**0.009**
									Log-egg	5.08	1	42.88	20.81	**<0.0001**
2013	9	63	1260	L (mm)	Dam	7.8	1	0.0053	Strain × log-egg	3.28	8	48.51	1.16	0.34
					Strain/Family	0.9	1	0.3	Strain × log-D	13.86	8	92.74	5.44	**<0.0001**
					Family/Rep	147.0	1	<0.0001	Log-D × log-egg	0.35	1	102.09	1.10	0.30
									Strain	6.05	8	93.24	5.44	**<0.0001**
									Log-D	0.43	1	102.40	2.64	0.11
									Log-egg	7.60	1	42.26	19.68	**0.0001**

One model was fitted per cohort. Random effects: Dam; maternal identity. Strain/Fam; family nested within strain (9–10, 6–7 and 4–9 families per strain in C2011, C2012 and C2013, respectively), Family/Rep; replicate nested within family (4, 3 and 2 replicates per family in C2011, C2012 and C2013, respectively). Chi.sq; value of the Chi square statistics. Chi.Df; the degrees of freedom for the test. P; p-value of the likelihood ratio test for the random effect. Fixed effects: Strain; Mowi (C2011–C2013), Mowi × Figgjo (C2011–2013), Figgjo (C2011–2013), Figgjo × Mowi (C2012–2013), Vosso (C2012–2013), Arna (C2012–2013), SalmoBreed (C2013), SalmoBreed × Vosso (C2013), Skibotn (C2013). Log-egg; mean family log egg diameter (mm). Log-D: median family replicate log degree-days post hatch. “Fixed effect” × “fixed effect”; two-way interaction between the respective fixed variables. Sum.sq; sums of squares. Num.Df; numerator degrees of freedom. Den.Df; denominator degrees of freedom (Satterthwaite's approximation) F; F-value. P; p-value. Significant fixed effects are marked in bold. For differences in least squares means between strains in C2013, see File S2.

An overall positive relationship between alevin length and median family replicate log degree-days post hatch were detected in C2011 and C2012 (Table 5). This relationship was of a weaker magnitude than the relationship between alevin length and egg size, i.e., alevin length increased with approximately 0.02 mm and 0.002 mm per 1 degree-day post hatch in C2011, and C2012, respectively. In C2013, a significant interaction between alevin length and median family replicate log degree-days post hatch was detected (Table 5). Thus, a general positive effect of degree-days post hatch was not detected in all strains, as the strains Figgjo, Figgjo × Mowi and Skibotn displayed a weak negative relationship between alevin length and degree days post hatch.

The effect of strain upon alevin length prior to the onset of exogenous feeding was not significant in two out of three cohorts (Table 5). In C2013 a significant effect of strain upon alevin length was detected. This was primarily due to the fact that the earliest hatching strain, Arna, that was terminated at a mean of 853 degree-days post hatch, displayed a significantly different alevin length in comparison to all other strains (except Skibotn), after removal of egg and degree-days post hatching effects (File S2).

Although significant effects of strain upon alevin growth were detected in one cohort of this study, growth rate prior to the onset of exogenous feeding did not differ between strains within the farmed/wild crosses, indicating similar utilisation of endogenous resources. This result is further supported by the fact no difference in alevin length was detected between maternal half-sibling strains, i.e., F_1_ hybrid strains and their wild or farmed counterpart, when investigated separately in models where only the respective half-sibling families were included in this pair-wise comparisons (data not presented).

## Discussion

The present study aimed to investigate embryonic development rate and alevin growth prior to the onset of exogenous feeding in Norwegian Atlantic salmon families of farmed, wild and hybrid origin. The experiment was based upon measurements taken from 131 families representing four wild, two farmed and three wild/farmed F_1_ hybrid strains. The main results can be summarised in the following two points: (i) Wild strains consistently hatched earlier than farmed strains, with F_1_ hybrid strains displaying intermediate hatching times. However, the absolute differences in cumulative degree-days to hatch between farmed and wild strains were very small. (ii) No significant effect of strain upon alevin length prior to the onset of exogenous feeding was detected in the wild/farmed crosses, although a positive effect of egg size upon alevin length was detected in salmon of all origins. Thus, based upon these results, it is concluded that additive genetic variation for embryonic development rate exists within Norwegian Atlantic salmon, although genetic differences between farmed and wild strains for this trait are small. It is further concluded that although directional selection for increased growth has resulted in farmed salmon displaying significantly higher growth rates than wild salmon under hatchery rearing conditions, this has not resulted in detectable differences in alevin growth prior to the onset of exogenous feeding in Norwegian Atlantic salmon of wild and farmed origin.

### Embryonic development rate

Detection of significant differences in cumulative degree-days to hatch in salmon of wild, farmed and hybrid origin is consistent with the result of previous studies demonstrating heritable differences in embryonic development rate in salmonids [45]–[47]. Even though actual differences in mean cumulative degree-days to hatch were small in this study, and thus not always statistically significant, they were nevertheless consistent across cohorts. Furthermore, F_1_ hybrids consistently displayed intermediate hatching times to the pure strains of the wild/farmed crosses. Also, reciprocal Figgjo × Mowi hybrids consistently displayed similar hatching times. This shows that reciprocal hybrids hatched at the same time even though they emerged from eggs potentially differing in both size and yolk composition, while they hatched at a different time in comparison to their maternal half-sibling strain, i.e., pure Figgjo or pure Mowi strain, that emerged from the same egg. Overall, these data indicate that there is additive genetic variation to embryonic development rate in Norwegian Atlantic salmon strains of wild and farmed origin. This supports an earlier study conducted on Canadian strains of Atlantic salmon [33].

Farmed alevins consistently hatched later than wild alevins in the wild/farmed crosses of this study. No indication of prolonged hatching times, i.e., increased trait variance, were observed in the farmed salmon. A positive relationship between time of hatch and time of emergence has been documented in salmonids [46], [48], and as early emerging alevins are likely to gain a competitive advantage due to prior residency [49]–[51], maladaptive expressions of this trait may result in reduced growth and decreased survival in the wild [30]. Thus, delayed emergence of farmed offspring could provide a barrier against genetic introgression. However, the small observed differences in developmental rates until hatch, i.e., less than 3 days between farmed and wild strains as reported here, are likely to be much less important towards time of emergence than the large in-river variation in spawning time documented in salmonids, which may be weeks and even months [31], [52], [53]. As spawning time is highly heritable in salmonids [54], and may have been altered by the process of domestication, it is important to also investigate timing of maturation, and thus spawning time, in adult salmon of farmed and wild origin.

Significant interactions between genotype and the environment in embryonic development rate have been documented in wild Atlantic salmon, where wild genotypes hatching early in a domestic environment were not the ones hatching early in a wild environment and *vice versa* [55]. Thus, in order to fully elucidate the extent of additive genetic variation and plasticity in embryonic development rate in Atlantic salmon of wild and farmed origin, comparative reaction norm studies, performed along an environmental gradient ranging from the farmed to the natural environment, and across different thermal regimes could provide further insights.

### Alevin growth prior to the onset of exogenous feeding

**Figure 6:**
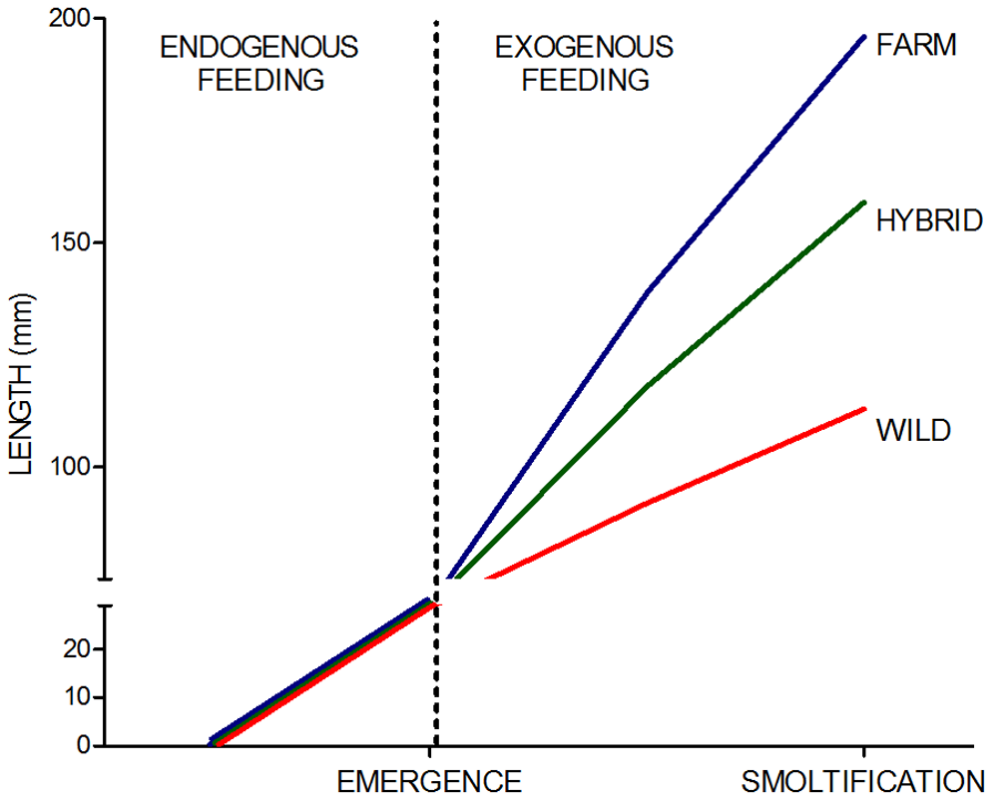
Growth of Norwegian Atlantic salmon prior to and post the onset of exogenous feeding. Additive genetic variation for growth is well documented in Norwegian Atlantic salmon of farmed, hybrid and wild origin, and farmed salmon outgrow wild salmon extensively at the smolt stage under hatchery conditions. However, this study demonstrates that ten generations of directional selection for increased growth has not resulted in significant differences in growth rate prior to the onset of exogenous feeding between farmed Norwegian salmon and its wild conspecifics. This figure is based upon observed growth measurements of the farmed Mowi strain, the wild Figgjo strain and their F_1_ hybrid strain of C2011 [18].

In general, a positive relationship between egg size and alevin length prior to the onset of exogenous feeding was detected in salmon families of all origins in this study. However, after removal of egg size effects, no significant effect of strain upon alevin length was detected in the wild/farmed crosses. Importantly, this included the half-sibling strains, where maternal effects were controlled for and the paternal contribution, i.e., the additive genetic variation, was isolated. Therefore, in general, genetic differences in alevin growth were not detected between salmon that had been farmed for approximately 10 generations, and their wild conspecifics. Nevertheless, in one year class, significant differences in length were detected between some of the wild strains, as well as between the wild Arna strains and all other strains, except Skibotn. This could indicate that there is a genetic component to early alevin growth. However, overall these data suggest that the documented difference in growth rate between wild and farmed Norwegian Atlantic salmon [19], [38], [56] are first detectable after the onset of exogenous feeding (Figure 6), and that utilisation of endogenous resources are similar in salmon of both origins.

This experiment was terminated close to complete yolk sac absorption, which marks the natural onset of exogenous feeding. All strains of the wild/farmed crosses were measured on the same degree-days post fertilisation. However, this was not possible for the wild strains of C2012 and C2013. Therefore, we cannot rule out that detection of strain-specific variation in alevin length, after removal of egg size effects, between the Arna wild strains in C2013 and the other strains may have been influenced by the small differences in the development stages at the time of sampling. However, significant differences in alevin length at yolk sac absorption has also been documented between two wild Canadian Atlantic salmon strains [57], [58], as well as between a wild and a Canadian strain domesticated for four generations [58]. In addition, significant differences were documented between wild strains and wild/farmed F_1_ and F_2_ hybrids and backcrosses [58]. When significant, wild strains were shorter than their farmed, hybrid and backcrossed conspecifics. Yolk sac conversion efficiency, i.e., utilisation of endogenous resources between time of hatch and time of emergence, were not significantly different between the wild and farmed strains, while some hybrid and backcrossed salmon displayed a significantly lower conversion efficiency relative to one of the wild salmon strains [58]. A recent study on Canadian Atlantic salmon also documented an increase, although not significant, in alevin length in offspring from farmed dams as compared to wild dams, while no difference in yolk conversion efficiency were detected [32]. Although there seems to be a genetic component to growth rate prior to the onset of exogenous feeding, together, the result of these studies suggest that the process of domestication has not resulted in any clear changes in early alevin growth and endogenous resource utilisation.

Maternal effects upon growth in the early life stages of salmonids may be strong [59], and egg size is considered as one of the most important maternal effects [60]. In the present study, alevins emerging from large eggs were in general larger at the onset of exogenous feeding than alevins emerging from small eggs, and this positive relationship between egg size and larvae size is well documented in salmonids [61]–[63] and across fish populations in general [66]. Maternal effects are however considered to be decreasing throughout yolk sac absorption [64]. For instance, small and late hatching alevins have been documented to have a higher growth rate than large and early hatching alevins, i.e., size dependent growth [65]. As there is a positive relationship between alevin size at emergence and survival [13], [67], [68], size dependent growth may be due to small and late hatching alevins maximising their growth potential prior to emergence [65]. A positive relationship between egg size and growth post emergence has been detected more than 100 days after emergence in salmon hatching in the wild [67], although no such relationship was detected in hatchery-reared salmon [69]. This indicates that the advantage of the maternal effect of egg size might be stronger when mortality, and thus selection, is intense. However, the selection pressure on early life history traits, such as survival in relation to egg size and time of emergence seems to vary in time and space, even at small scales [70], [71], and furthermore to be influenced by inter-species competition in nature [72]. In addition, emergence at differing developmental stages, in relation to temperature, has been documented, i.e., smaller alevins emerged with a larger remaining yolk sac at low temperatures, than their siblings at high temperatures, illustrating phenotypic plasticity of body size at emergence [73].

A positive relationship between degree-days post hatch and alevin length at termination was detected in this study. However, in one cohort, a negative relationship between time post hatch and alevin size was detected in some of the strains. This could indicate that strains in this study hatched at differing developmental stages, potentially masking any strain differences in growth rate, as late hatching alevins may have compensated by displaying increased growth rates up until the onset of exogenous feeding [66]. Strain variation in growth rate prior to the onset of exogenous feeding could also be masked by transgenerational environmental effects, such as difference in yolk composition between egg from parents reared in a farmed environment or from the wild. Thus, comparing growth of alevins emerging from sized matched egg of parents having experienced the same ambient conditions could be useful, in order to investigate the full extent of additive genetic variation in somatic growth rate prior to the onset of exogenous feeding in Atlantic salmon of wild and farmed origin.

## Supporting Information

File S1 **Model selection, model 1.**
(XLS)

File S2 **Differences of least squares means, models 2 and 3.**
(XLS)

File S3 **Data set.**
(XLS)
