# Correction: Hatching time and alevin growth prior to the onset of exogenous feeding in farmed, wild and hybrid Norwegian Atlantic salmon

**DOI:** 10.1371/journal.pone.0118419

**Published:** 2015-03-06

**Authors:** The PLOS ONE Staff

In the Methods section, there is an error in the third equation in the section titled “Statistical Analysis.” The variable *b*_*da*_ *m* is incorrect. It should read *b*_*dam*_. Please view the complete, correct equation here:

$$L=\alpha +\beta_{1}E+\beta_{2}S+\beta_{3}D+\beta_{4}ES+\beta_{5}ED+\beta_{6}SD+b_{dam}+b_{f(S)}+b_{r(f)}+\varepsilon$$

In Table 2, the values within the CV column under the “Length prior to start feeding (mm)” header are formatted incorrectly. Please see the corrected Table 2 here.

**Table 2:** Number, egg size, cumulative degree-days to hatch and total length prior to the onset of exogenous feeding in salmon of farmed, hybrid and wild origin of C2011, C2012 and C2013.

					Egg diameter (mm)			Cumulative degree-days to hatch				Not hatched		Length prior to start-feeding (mm)				
Cohort	Strain	Origin	Families	n	Median	Mean	Range	Median	Mean	Range	CV	n	%	n	Median	Mean	Range	CV
2011	Figgjo	Wild	9	900	5.7	5.78	5.3–6.1	555	557	534–590	1.22	0	0	360	28.7	28.7	26.4–30.8	2.85
	Mowi x Figgjo	F1 hybrid	10	1000	6	6	5.8–6.3	563	561	521–590	1.63	0	0	400	28.9	29	26.6–30.8	2.42
	Mowi	Farm	10	1000	6	5.97	5.8–6.1	563	564	525–601	1.73	29	2.9	400	29	29	26.2–31.5	2.49
2012	Figgjo	Wild	6	451	6.25	6.26	6.0–6.4	550	551	538–573	1.55	3	0.7	180	28.2	28.3	25.7–30.3	2.83
	Figgjo x Mowi	F1 hybrid	6	450	6.2	6.2	6.0–6.4	556	556	527–619	1.68	3	0.7	180	28.3	28.2	26.6–29.9	2.35
	Mowi x Figgjo	F1 hybrid	7	525	6.1	6.06	5.8–6.4	556	554	538–585	1.21	4	0.8	210	28.1	28.1	25.6–30.0	3.31
	Mowi	Farm	7	525	6.1	6.14	5.8–6.3	556	558	515–590	1.77	3	0.6	210	28.2	28.1	25.4–29.9	2.71
	Vosso	Wild	7	526	6	5.8	5.3–6.3	559	558	523–576	1.18	1	0.2	210	27.4	27.4	25.3–29.5	2.97
	Arna	Wild	6	450	5.7	5.72	5.4–6.1	553	553	537–576	1.22	3	0.7	180	28.4	28.4	26.9–30.3	2.92
2013	Figgjo	Wild	9	540	6	5.86	5.0–6.3	551	552	514–587	1.87	4	0.7	180	27.7	27.8	25.1–30.1	3.48
	Figgjo x Mowi	F1 hybrid	7	420	6	5.89	5.2–6.3	557	556	524–599	1.75	1	0.2	140	28	27.9	25.2–30.0	3.63
	Mowi x Figgjo	F1 hybrid	9	540	6.1	6.12	5.7–6.4	557	559	539–599	1.56	3	0.6	180	28.6	28.5	25.1–30.6	3.24
	Mowi	Farm	8	480	6.1	6.11	5.8–6.4	557	563	489–677	2.61	4	0.8	160	28.4	28.3	25.2–30.8	3.7
	Vosso	Wild	4	240	5.9	5.88	5.7–6.0	556	559	544–592	1.6	2	0.8	80	28.3	28.3	26.5–30.0	2.61
	SalmoBreed x Vosso	F1 hybrid	6	360	6	6.03	5.8–6.3	562	558	501–604	1.65	17	4.7	120	28.1	28	25.9–29.4	2.15
	SalmoBreed	Farm	8	480	6.1	6.1	5.8–6.4	562	561	519–675	2.03	10	2.1	160	28.2	28.2	26.7–29.9	2.38
	Arna	Wild	8	480	5.85	5.91	5.6–6.3	553	548	507–577	2.3	1	0.2	160	27.5	27.6	24.7–30.2	3.85
	Skibotn	Wild*	4	240	6.25	6.25	6.1–6.4	559	558	515–607	1.94	7	2.9	80	27.8	27.6	24.7–29.1	3.76

CV = coefficient of variation.

*Wild salmon population reared in freshwater at the Norwegian Gene Bank for Atlantic salmon.
